# Association of Weight Fluctuation With Mortality in Japanese Adults

**DOI:** 10.1001/jamanetworkopen.2019.0731

**Published:** 2019-03-15

**Authors:** John Cologne, Ikuno Takahashi, Benjamin French, Akiko Nanri, Munechika Misumi, Atsuko Sadakane, Harry M. Cullings, Yuko Araki, Tetsuya Mizoue

**Affiliations:** 1 Department of Statistics, Radiation Effects Research Foundation, Hiroshima, Japan; 2 Department of Clinical Studies, Radiation Effects Research Foundation, Hiroshima, Japan; 3 Department of Food and Health Sciences, International College of Arts and Sciences, Fukuoka Women's University, Fukuoka, Japan; 4 Center for Clinical Sciences, Department of Epidemiology and Prevention, National Center for Global Health and Medicine, Tokyo, Japan; 5 Department of Epidemiology, Radiation Effects Research Foundation, Hiroshima, Japan; 6 Graduate School of Integrated Science and Technology, Department of Informatics, Shizuoka University, Shizuoka, Japan

## Abstract

**Importance:**

Weight cycling is associated with the risk of mortality from heart disease, but many studies have not distinguished between simple nonlinear (monotone) weight changes and more complex changes that reflect fluctuations.

**Objective:**

To assess whether extreme body weight variation is associated with mortality after controlling for nonlinear weight changes.

**Design, Setting, and Participants:**

In this prospective clinical cohort study, 4796 Japanese atomic bomb survivors were examined in the clinic as part of a biennial health examination and research program. The study consisted of a 20-year longitudinal baseline period (July 1, 1958, to June 30, 1978) and subsequent mortality follow-up of 27 years (July 1, 1978, to June 30, 2005) Participants were initially between the ages of 20 and 49 years during the baseline period and, throughout the baseline period, had no diagnoses of cardiovascular disease (CVD) or cancer and attended at least 7 of 10 scheduled examinations. Data analysis was performed from October 16, 2015, to May 13, 2016.

**Exposures:**

Residual variability in body mass index (BMI) during the baseline period.

**Main Outcomes and Measures:**

Outcomes were mortality from ischemic heart disease, cerebrovascular disease, other CVDs combined, other causes (except cancer), and cancer. Root mean squared error was calculated to capture individual residual variation in BMI after adjustment for baseline BMI trends, and the association of magnitude of residual variation with mortality was calculated as relative risk.

**Results:**

In total, 4796 persons (mean [SD] age, 35.0 [7.3] years at first baseline examination; 3252 [67.8%] female; mean [SD] BMI, 21.2 [2.8] at first baseline visit [20.6 (2.4) among men and 21.5 (2.9) among women]) participated in the study. During follow-up, 1550 participants died: 82 (5.3% of all deaths) of ischemic heart disease, 181 (11.7%) of cerebrovascular disease, 186 (12.0%) of other CVDs, 615 (39.7%) of cancer, and 486 (31.3%) of other causes. Magnitude of residual variation in weight was associated with all-cause mortality (relative risk, 1.25 for 1 U of additional variation; 95% CI, 1.06-1.47) and ischemic heart disease mortality (relative risk, 2.49; 95% CI, 1.41-4.38).

**Conclusions and Relevance:**

The findings suggest that an association exists between weight variation and heart disease mortality and that weight loss interventions, if deemed to be necessary, should be considered carefully.

## Key Points

**Question:**

Are weight fluctuations associated with mortality after controlling for simple nonlinear weight changes?

**Findings:**

In this cohort study of 4796 Japanese adults, after adjustment for simple nonlinear change in body mass index, a stronger association was found between residual body mass index variation (fluctuation) and all-cause mortality and ischemic heart disease mortality.

**Meaning:**

The findings suggest that when advising weight loss in overweight or obese patients, physicians should consider not only the transient results of weight loss interventions but also the potential long-term health consequences of weight fluctuation.

## Teaser

This cohort study of Japanese adults who survived the atomic bomb assesses whether extreme body weight variation is associated with mortality after controlling for nonlinear weight changes.

## Introduction

Weight cycling (fluctuation) can occur when overweight or obese adults try to lose weight. Extreme weight fluctuation has been associated with higher risk of morbidity and mortality,^1,2,3,4,5,6^ leading to questions about the prudence of recommending weight loss.^7^ However, some studies^7,8,9,10^ have found no effects of weight fluctuation. Weight fluctuation affects the immune system, glucose tolerance, and inflammation, which are associated with metabolic and cardiovascular diseases (CVDs) and cancer,^11,12,13^ and might be associated with clinically relevant lowering of high-density lipoprotein cholesterol levels.^14^ It is therefore important to better understand the health consequences of weight fluctuation, whether intentional or unintentional.

Simple methods used to capture weight fluctuation in previous studies^1,2,3,4,6,15^ had shortcomings. First, the coefficient of variation or residual root mean squared error (RMSE) derived from linear regressions^1,2,3^ cannot distinguish fluctuations from simple nonlinear change in weight. Second, approaches based on total change or sum of deviations from an overall mean^4^ are sensitive to the number of measurements and length of time. Third, categorizing participants into a small number of a priori weight-trajectory classes^4,6^ or data-determined latent classes^15^ does not provide a quantitative measure of weight variability.

A reliable method to assess and quantify weight fluctuation is therefore needed to better assess how mortality risk is associated with weight fluctuation. We used a flexible statistical modeling approach to fit trajectories of body mass index (BMI) (calculated as weight in kilograms divided by height in meters squared) by using polynomial functions with hierarchical (mixed-effects or random-coefficient) models. The resulting RMSE of residuals does not include variation attributable to simple nonlinearities in BMI trajectories but captures variation attributable to large fluctuations in BMI, including cycling. We then quantified the association between residual variation and subsequent disease mortality.

## Methods

### Study Design and Participants

The source cohort was the Adult Health Study, a prospective program of biennial clinical examinations among Japanese atomic bomb survivors that was begun in 1958.^16^ The study consisted of a 20-year longitudinal baseline period (July 1, 1958, to June 30, 1978) and subsequent mortality follow-up of 27 years (July 1, 1978, to June 30, 2005). Data analysis was performed from October 16, 2015, to May 13, 2016. Candidate participants were 20 through 49 years of age at the time of their first clinical examination and attended at least 7 of 10 possible examinations during a 20-year baseline period until 1978. Seven was selected as the minimum number of examinations attended because assessing fluctuation requires a large number of longitudinal observations. Mortality follow-up began with the first clinical examination attended after 1978 and was based on nationwide death registrations.^17^ Participants were excluded if they died or had CVD (ischemic heart disease and stroke) or cancer during the baseline period. Cardiovascular disease was identified through clinical examinations, with *International Classification of Diseases* codes as defined in eTable 1 in the Supplement. Cancer was identified through local cancer registries.^18^ The study was reviewed and approved by the Human Investigation Committee of the Radiation Effects Research Foundation, Hiroshima, Japan, and oral informed consent was obtained in accordance with legal requirements for human research in Japan. Data were deidentified. In preparing this report, we followed the Strengthening the Reporting of Observational Studies in Epidemiology (STROBE) reporting guideline for observational cohort studies.

Steps of participant selection for the study are given in eFigure 1 in the Supplement. Starting with 17 396 cohort members, we excluded any who had cancer or CVD or who died before the end of the baseline period (1979), leaving 13 929 members. Among these, we further excluded any who had fewer than 7 examinations during the baseline period, were outside the age range of 20 to 49 years at their first examination, or had at most 1 recorded BMI measurement during the baseline period despite attending at least 7 examinations. The total number of eligible participants for baseline BMI trajectory analyses was therefore 4796.

For follow-up analyses, we excluded 819 participants who were missing estimated radiation dose and an additional 30 who were missing smoking status (4 participants were missing both), leaving 3947 participants eligible for follow-up analysis. Follow-up began with the first clinical examination attended during the follow-up period and continued until first diagnosis of CVD or cancer, death, or the end of 2005, whichever came first. Among participants who were eligible for follow-up analysis, 168 never attended an examination during the follow-up period and thus did not have a bona fide date for start of follow-up. Many of these individuals died during the first few years of the follow-up period and thus could have had health issues associated with unintentional weight loss during the latter part of the baseline period. Others were assumed to have implicitly revoked their consent. For these reasons, those 168 persons were excluded from the follow-up analyses, leaving 3779 participants (attendees) for the follow-up analysis.

### Statistical Analysis

The statistical analysis was conducted in 2 stages. In the first stage, we fit polynomial mixed models in age to estimate smooth trends in baseline period BMI data and derived estimates of residual variation (RMSE of differences between observed and fitted BMI). In the second stage, we used the residual variation values computed in the first stage to assess the risk of subsequent mortality during the follow-up period by fitting Cox proportional hazards regression models. This procedure was performed using both continuous total variation within each participant and categories (quintiles) of total variation.

Statistical analyses were conducted using Stata, version 14 (StataCorp). Longitudinal baseline trajectories were fit using multilevel longitudinal modeling with restricted maximum likelihood estimation.^19^ Follow-up analyses were based on the Kaplan-Meier method and Cox proportional hazards regression.^20^ *P* values are based on likelihood ratio tests and are 2-sided, with significance set at the 5% level; 95% CIs are based on Wald statistics.

#### BMI Trajectories

We analyzed BMI rather than weight because residuals about BMI trajectories are standardized to height, whereas the scale of residuals based on weight depends on height. Baseline BMI trajectories were fitted using mixed-effects (random-coefficient) regression models,^21,22^ with linear, linear-quadratic, third-degree (linear, quadratic, and cubic [LQC]), and fourth-degree (linear, quadratic, cubic, and quartic [LQCQ]) polynomials in longitudinal age at measurement (centered at 44 years). Fitted trajectories were adjusted for age at entry (centered at 35 years) and sex. Additional covariates potentially related to BMI were not adjusted because the purpose was to adjust for broad trends in BMI not to predict or explain those trends. Random effects were included for the intercept and all parameters of longitudinal age in the trajectory (eMethods in the Supplement). Sex was also included in the random linear term of the trajectory model to accommodate interaction between sex and longitudinal age. Within-subject residuals and random effects were assumed to be normally distributed. We computed the residual RMSE within each individual as the square root of the variance of his or her residuals (observed minus fitted values of BMI at each BMI measurement time point) by using fitted values based on the trajectory model. We also examined whether there was an association between radiation exposure and BMI trajectories by including radiation dose as an additional covariate in the trajectory model, using 3977 baseline participants with known radiation dose.

#### Mortality Risk

Follow-up for mortality risk analysis began at the time of the first clinical examination attended during the follow-up period. Individual causes of death were classified by *International Classification of Diseases* code as listed in eTable 2 in the Supplement. Association of weight variation with mortality was assessed using univariate (crude) log rank, Wilcoxon, and trend tests for survival stratified on population quintiles of RMSE and multivariable Cox proportional hazards regression analysis using individual values of continuous RMSE or RMSE quintiles. Cox proportional hazards regressions were fit with follow-up age as the primary time scale and left truncation on age at the start of follow-up^23^ and were stratified on city of residence and sex. Participants still alive at the end of the follow-up period (June 30, 2005) were treated as censored.

Cox proportional hazards regression models are defined in the eMethods in the Supplement. In the Cox proportional hazards regression analysis, we adjusted for year of birth (centered at 1924), mean height during the baseline period (centered at 161.5 cm for men and 160.0 cm for women), an indicator of having ever smoked, and radiation dose to the colon (in weighted grays, with weights 1 for γ and 10 for neutron components). We adjusted for height because BMI alone might not capture all the information on body size.^24^ We also adjusted for overall BMI and overall linear trend in BMI during the baseline period using the random effects estimated from a linear random-coefficient trajectory model. Because overall weight gain and overall weight loss have different clinical significance, we adjusted separately for overall increase (subject-specific random slope >0) and overall decrease (slope <0). Both trend variables were multiplied by 10 to estimate the association with a unit change in BMI during 10 years. Interactions between birth year and city, between radiation dose and the 4 BMI-related variables (overall BMI level, RMSE, and overall gain or loss), and between ever-smoked and sex were assessed by adding cross-products of the corresponding variables to the Cox proportional hazards regression model. We used a simple baseline ever-never variable for smoking status because of the potentially complex association between smoking and obesity in terms of their association with CVD mortality.^25^ Risk of having diabetes diagnosed before the start of follow-up was also examined in the Cox proportional hazards regression model. The proportional hazards assumption was investigated using Schoenfeld residual plots.

## Results

### Characteristics of Study Participants

In total, 4796 persons (mean [SD] age, 35.0 [7.3] years at first baseline examination; 3252 [67.8%] female; mean [SD] BMI, 21.2 [2.8] at first baseline visit [20.6 (2.4) among men and 21.5 (2.9) among women]) participated in the study. No notable difference in terms of mean BMI was found between included participants who attended 7 or more examinations during the baseline period and otherwise eligible persons who attended fewer than 7 examinations during the baseline period (eTable 3 in the Supplement). Among the 3779 follow-up participants, 2582 (68.3%) were women and 1197 (31.7%) were men. On the basis of mean BMI during baseline (intercept of the mixed model), 3175 follow-up participants had normal weight (BMI ≤25.0), 545 were overweight (BMI >25.0), and 59 were obese (BMI >30.0).

No striking difference was found between those who attended a clinical examination during the follow-up period (attendees) and those who did not. The mean (SD) age at the start of the baseline period was 35.0 (7.4) years among the 3779 attendees and 37.3 (8.0) years among the 168 who did not attend, the mean BMI (SD) during the baseline period was 22.1 (3.0) in both groups, and there was little difference in the proportions according to sex or city of residence (eTable 4 in the Supplement). Although 2229 attendees (59.0%) were alive at the end of the follow-up period, only 61 (36.3%) of those who did not attend were alive at the end of the follow-up period; in fact, many of the nonattendees died during the first few years of the follow-up period (eFigure 2 in the Supplement).

During the follow-up period, 1550 participants (41.0%) died: 82 (5.3% of all deaths) of ischemic heart disease, 181 (11.7%) of cerebrovascular disease, 186 (12.0%) of other CVDs, 615 (39.7%) of cancer, and 486 (31.3%) of other causes. Demographic details of individual causes of death are given in eTable 5 in the Supplement.

### Baseline BMI Trajectories

With a linear mixed-effects model, the estimated baseline BMI trajectory intercept coefficient was 21.5 (95% CI, 21.3-21.6) (Table 1), and the linear BMI coefficient was increased overall by 0.082 (95% CI, 0.076-0.089) per year (Table 1). Sex and age at entry (start of the baseline period), as well as an interaction between sex and longitudinal age, were important variables. There was notable interindividual heterogeneity (variation in random effects) in both intercepts and slopes, with a correlation of 0.36 (95% CI, 0.33-0.39) between the intercept and slope random effects.

**Table 1.** Estimated Baseline BMI Trajectory Coefficients, Adjusted for Sex and Age at Entry

Model	Coefficient (95% CI)^a^					AIC	BIC
	Intercept	Linear	Quadratic	Cubic	Quartic		
Linear	21.5 (21.3 to 21.6)	0.082 (0.076 to 0.089)	NA	NA	NA	144 664.7	144 742.9
Linear and quadratic	21.5 (21.4 to 21.6)	0.081 (0.074 to 0.088)	−0.0016 (−0.0019 to −0.0014)	NA	NA	142 659.3	142 772.2
Linear, quadratic, and cubic (no correlations among random effects)^b^	21.4 (21.3 to 21.6)	0.087 (0.080 to 0.095)	−0.0020 (−0.0023 to −0.0017)	−7.3 × 10^−5^ (−9.0 × 10^−5^ to −5.6 × 10^−5^)	NA	143 876.9	143 981.1
Linear, quadratic, cubic, and quartic (no correlations among random effects)^b^	21.5 (21.3 to 21.6)	0.086 (0.079 to 0.094)	−0.0036 (−0.0040 to −0.0032)	−8.4 × 10^−5^ (−1.0 × 10^−4^ to −6.6 × 10^−5^)	6.2 × 10^−6^ (5.1 × 10^−6^ to 7.2 × 10^−6^)	143 644.7	143 766.2

Abbreviations: AIC, Akaike information criterion; BIC, Bayesian information criterion; BMI, body mass index (calculated as weight in kilograms divided by the height in meters squared); NA, not applicable.

^a^ Coefficients represent values for men aged 35 years at start of baseline.

^b^ The linear-quadratic-cubic and linear-quadratic-cubic-quartic models did not converge when an unstructured covariance matrix was used; thus, an assumption of no correlations among random effects was used for those models.

Fitted trajectory parameters with the addition of higher-order terms (quadratic, cubic, and quartic) are also given in Table 1. The Figure shows linear and LQC mixed-model fitted trajectories for 2 selected participants along with the fit of an ordinary, individual-participant, fourth-order polynomial least-squares regression. The random-coefficient models ignored extreme fluctuations, leaving such fluctuations to the estimate of residual variation, whereas ordinary polynomial regression left little residual variation. Straight-line regression was not adequate to remove simple (noncycling) patterns of weight change over time, whereas the higher-order random-coefficient models removed simple nonlinearities. Thus, the linear model produced the largest RMSE value, and the RMSE value from the LQC mixed model was between that of the linear model and the ordinary LQCQ regression model (Figure). Although goodness-of-fit criteria (Akaike information criterion and Bayesian information criterion) suggested that the LQCQ mixed model provided the best fit among the random-coefficient models, there was little practical difference between the fits of the LQC and LQCQ mixed models. We therefore used RMSE based on the LQC model in the follow-up analyses. There was no suggestion of an association between radiation dose and overall BMI level (estimated coefficient for radiation dose, −0.065; 95% CI, −0.24 to 0.11; *P* = .46), and residuals from LQC models fit with or without adjustment for radiation dose were nearly identical (mean [SD] of residuals with adjustment for radiation dose: 0.112 [2.479]; range, −39.369 to 32.924; mean [SD] of residuals without adjustment for radiation dose: 0.112 [2.479]; range, −39.372 to 32.924).

**Figure.**
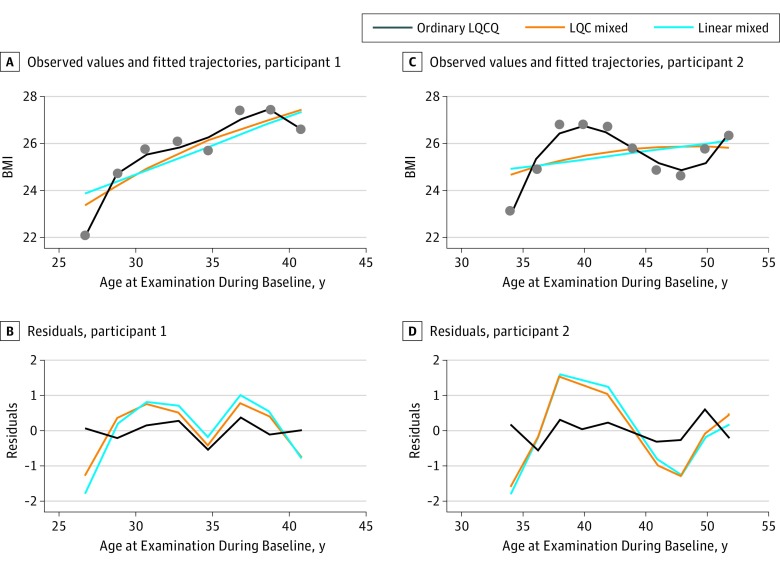
Observed and Fitted Values of Body Mass Index (BMI) and Residuals From 2 Selected Participants A and C, Observed values of BMI (calculated as weight in kilograms divided by the height in meters squared) and fitted trajectories (lines). B and D, Residuals (observed minus fitted values, unstandardized). LQC indicates linear, quadratic, and cubic; LQCQ, linear, quadratic, cubic, and quartic.

### Mortality Risk

Of 1550 deaths, numbers (percentages [crude rates per 10 000 person-years]) by cause were as follows: ischemic heart disease, 82 (5.3% [9.8 × 10^−4^]); cerebrovascular disease, 181 (11.7% [21.6 × 10^−4^]); other CVD, 186 (12.0% [22.2 × 10^−4^]); cancer, 615 (39.7% [73.4 × 10^−4^]); and other causes, 486 (31.6% [58.0 × 10^−4^]). Numbers of deaths due to all causes are given in Table 2 and stratified by RMSE quintile. Also given in Table 2 are conversions from BMI RMSE to residual RMSE for body weight for a 1.7-m-tall individual. A 1.0-U of residual BMI RMSE corresponds to a residual weight RMSE of 2.89 kg for a 1.7-m-tall individual (787 participants [16.4%] had BMI RMSE >1.0), and the maximum observed value of BMI RMSE (4.085) corresponds to a weight RMSE of 11.8 kg for a 1.7-m-tall individual. To interpret the RMSE measure, note that individuals in the highest quintile might have several cycles of BMI changes of 2 to 3 U (eFigure 3 in the Supplement), which amounts to repeated loss and gain of approximately 6 to 9 kg in body weight during each cycle.

**Table 2.** Numbers of Deaths From All Causes by Quintile of Residual BMI Variability (RMSE) Based on Linear-Quadratic-Cubic Model for Baseline BMI Trajectory

Variable	BMI RMSE Quintile					
	0.113-0.451	0.452-0.587	0.588-0.721	0.722-0.939	0.940-4.085	Total
Weight RMSE for a 1.7-m-tall individual, range, kg	0.327-1.30	1.31-1.70	1.70-2.08	2.09-2.71	2.72-11.8	NA
Died during follow-up, No. (%)						
No	454 (61.8)	434 (57.0)	453 (63.1)	447 (56.8)	441 (56.7)	2229 (59.0)
Yes	280 (38.2)	328 (43.0)	265 (36.9)	340 (43.2)	337 (43.3)	1550 (41.0)
Total	734	762	718	787	778	3779

Abbreviations: BMI, body mass index (calculated as weight in kilograms divided by the height in meters squared); NA, not applicable; RMSE, root mean squared error.

Crude survival functions did not reveal any important association with RMSE, but after sex was accounted for, participants in the highest quintile of RMSE had the lowest survival probabilities (eFigure 4 in the Supplement). With adjustment for other covariates, continuous RMSE was associated with total mortality (relative risk [RR], 1.25; 95% CI, 1.06-1.47) (Table 3). The association between RMSE and total mortality was independent of BMI class (as judged by mean BMI during the baseline period, the intercept of the fitted LQC model): among 3175 persons with normal BMI (≤25.0), the RR was 1.21 (95% CI, 1.03-1.44), and among 604 persons who were overweight or obese (BMI >25.0), the RR was 1.35 (95% CI, 1.09-1.66) (likelihood ratio test of the interaction *P* = .28). No evidence of an association was found between overall weight gain and total mortality, but a detrimental association was found between overall weight loss and total mortality. This association remained when follow-up was left-truncated at 2 or 5 years after the start of follow-up to account for possible reverse causation (Table 3). No interaction with RMSE was found for overall BMI level, BMI gain, BMI loss, or smoking. Having diabetes before the start of the follow-up period was associated with mortality risk, but diabetes was not confounded with other variables for other factors. There was evidence of heterogeneity among RMSE quintiles in terms of total mortality (Table 4), and the highest RMSE quintile was associated with total mortality. A nearly linear increase with RMSE was observed when RMSE was categorized into 20 equal-sized groups (eFigure 5 in the Supplement). No interactions were detected between radiation dose and any of the BMI-related factors.

**Table 3.** Relative Risks (95% CIs) From Cox Proportional Hazards Regression Model Fits to Continuous Residual BMI Variability (RMSE) Based on Linear-Quadratic-Cubic Baseline BMI Trajectories

Mortality End Point^a^ (No. of Deaths)	Relative Risk (95% CI)					
	RMSE (BMI Difference of 1)	BMI (BMI Difference of 1)	BMI Gain (BMI Increase of 1 During 10 y vs No Gain)	BMI Loss (BMI Decrease of 1 During 10 y vs No Loss)	Ever Smoker (vs Never Smoker)	Radiation (Dose Difference of 1 Gy)
All causes						
From start of follow-up (1550)	1.25 (1.06-1.47)	1.02 (1.00-1.04)	0.97 (0.90-1.03)	1.36 (1.16-1.59)	2.53 (1.85-3.45)	1.30 (1.19-1.42)
With 2-y truncation (1497)	1.22 (1.04-1.44)	1.03 (1.01-1.05)	0.97 (0.91-1.04)	1.35 (1.15-1.59)	2.53 (1.84-3.48)	1.30 (1.19-1.42)
With 5-y truncation (1414)	1.26 (1.06-1.49)	1.02 (1.00-1.05)	0.96 (0.90-1.03)	1.34 (1.31-1.58)	2.49 (1.80-3.43)	1.29 (1.17-1.41)
With adjustment for diabetes during baseline	1.25 (1.07-1.47)	1.01 (0.99-1.03)	0.97 (0.91-1.04)	1.32 (1.13-1.54)	2.52 (1.84-3.44)	1.30 (1.20-1.42)
Ischemic heart disease (82)	2.49 (1.41-4.38)	1.08 (1.00-1.17)	0.76 (0.57-1.02)	0.57 (0.27-1.23)	2.62 (0.62-11.1)	1.27 (0.85-1.88)
Cerebrovascular disease (181)	0.89 (0.53-1.47)	1.11 (1.05-1.17)	0.87 (0.72-1.05)	0.95 (0.57-1.57)	1.15 (0.54-2.46)	0.94 (0.69-1.28)
Other CVD (186)	1.46 (0.92-2.30)	1.03 (0.98-1.09)	0.80 (0.65-0.97)	1.01 (0.63-1.62)	2.17 (0.80-5.88)	1.35 (1.04-1.75)
Cancer (615)	0.98 (0.75-1.30)	1.02 (0.99-1.05)	1.05 (0.95-1.16)	1.34 (1.01-1.76)	4.10 (2.28-7.36)	1.42 (1.26-1.61)
Other causes (486)	1.49 (1.14-1.95)	0.98 (0.94-1.01)	1.00 (0.89-1.13)	1.84 (1.45-2.33)	2.16 (1.29-3.60)	1.24 (1.06-1.46)

Abbreviations: BMI, body mass index (calculated as weight in kilograms divided by the height in meters squared); CVD, cardiovascular disease; RMSE, root mean squared error.

^a^ Models included stratification on city and sex and adjustment for birth year, height, ever smoked, and sex  × ever-smoked interaction.

**Table 4.** Relative Risks (95% CIs) From Cox Proportional Hazards Regression Model Fits to Quintiles of Residual BMI Variability (RMSE) From Linear-Quadratic-Cubic Baseline BMI Trajectories

Mortality End Point^a^	Relative Risk (95% CI) by BMI RMSE Quintile^b^				*P* Value for Heterogeneity Test
	0.452-0.587	0.588-0.721	0.722-0.939	0.940-4.085	
All causes	1.10 (0.94-1.30)	1.07 (0.90-1.27)	1.10 (0.93-1.30)	1.31 (1.10-1.56)	.04
Ischemic heart disease	1.27 (0.54-2.96)	1.67 (0.74-3.80)	2.06 (0.92-4.61)	3.27 (1.47-7.31)	.03
Cerebrovascular disease	1.14 (0.70-1.85)	1.04 (0.63-1.72)	0.91 (0.55-1.51)	1.02 (0.61-1.73)	.92
Other CVD	1.46 (0.88-2.44)	1.34 (0.80-2.27)	1.56 (0.93-2.63)	1.75 (1.03-3.00)	.31
Cancer	1.01 (0.79-1.29)	0.94 (0.73-1.22)	0.94 (0.72-1.23)	1.03 (0.78-1.36)	.94
Other causes	1.10 (0.82-1.49)	1.12 (0.83-1.52)	1.17 (0.86-1.60)	1.51 (1.10-2.06)	.11

Abbreviations: BMI, body mass index (calculated as weight in kilograms divided by the height in meters squared); CVD, cardiovascular disease; RMSE, root mean squared error.

^a^ Models included stratification on city and sex and adjustment for birth year, height, ever smoked, and sex × ever-smoked interaction and radiation dose.

^b^ The lowest BMI RMSE quintile (0.1128-0.4521) was the reference category (no individuals had BMI RMSE values <0.1128).

Among individual causes of death, RMSE was associated with death from ischemic heart disease with continuous RMSE (Table 3) and in the highest RMSE quintile (RR for ischemic heart disease, 3.27; 95% CI, 1.47-7.31) (Table 4). Deaths from cerebrovascular disease or cancer were not associated with RMSE. Although continuous RMSE was not associated with other CVD causes and heterogeneity among RMSE quintiles was not apparent with other CVD causes (Table 4), the risk of continuous RMSE with other CVD causes of death was large, and all quintiles had high RRs compared with the lowest quintile (0.452-0.587 quintile, 1.46 [95%CI, 0.88-2.44]; 0.588-0.721 quintile, 1.34 [95% CI, 0.80-2.27]; 0.722-0.939 quintile, 1.56 [95% CI, 0.93-2.63]; and 0.940-4.085 quintile, 1.75 [95% CI, 1.03-3.00]). Associations of RMSE with deaths from other causes were qualitatively and quantitatively similar to those for all-cause mortality (0.452-0.587 quintile, 1.10 [95% CI, 0.94-1.30]; 0.588-0.721 quintile, 1.07 [95% CI, 0.90-1.27]; 0.722-0.939 quintile, 1.10 [95% CI, 0.93-1.30]; and 0.940-4.085 quintile, 1.31 [95% CI, 1.10-1.56]).

## Discussion

Obesity is linked not only to increased risk of heart disease morbidity and mortality^6,26^ but also to a loss of years of life spent in good health.^27^ The increase in obesity worldwide has led to interest in interventions to reduce risk of adverse health outcomes,^28^ with the traditional approach being weight loss or obesity treatment.^29,30^ However, evidence is weak that intentional weight loss reduces risk^31^; weight loss may be associated with increased risk of mortality,^32^ and clinical misconceptions about obesity persist.^33^ Nevertheless, recommending weight loss remains a common strategy for managing the overweight and obesity epidemic. Although some individuals may achieve sustained weight loss,^34^ unsuccessful attempts to lose weight are common and may result in repeated cycles of weight loss and gain or extreme weight variability.^35^ Whether such weight fluctuation is associated with greater risk of mortality remains controversial.^33^

Previous work^1,2,3,4,6,15^ on the association of weight variability and mortality has been based on simple statistical approaches that may be contaminated by nonlinear changes in weight not reflecting extreme variation, such as cycling. The present work improves on previous methods, and the findings suggest that mortality risk is associated with weight fluctuation. In our analysis, total mortality and ischemic heart disease mortality during the follow-up period were associated with large residual variation in BMI during the baseline period. Cerebrovascular disease mortality was not associated with residual BMI variation, but there was suggestive evidence of an association between large residual variation and other CVD mortality. Cancer mortality was not associated with residual BMI variation, consistent with previous findings.^35^ Our estimated RRs for total mortality by increasing quintile of RMSE (1.10, 1.07, 1.10, and 1.31 compared with the lowest quintile) were comparable to or higher than those for Japanese American men based on quintiles of weight variation from simple linear regressions reported by Iribarren at al^2^ (1.14, 1.07, 1.01, 1.25). In addition, their reported RRs of death in the highest quintile of weight variation included 1.29 for coronary heart disease and 1.41 for all cardiovascular causes, whereas we found RRs of 3.27 for ischemic heart disease and 1.75 for all cardiovascular causes other than ischemic heart disease or cerebrovascular disease.

The findings suggest that use of mixed-effects (random-coefficient) regression models is better suited than ordinary regression modeling for studying weight fluctuation. Using polynomial models avoids including in the estimate of residual variation the contribution from broad nonlinear changes in weight, but with mixed-effects models, the interindividual variation in trajectory shapes is less than that obtained by fitting individual polynomial trajectories with ordinary regression because the mixed-model approach performs population-based smoothing.^36^ Similar to Lissner et al,^1^ we included the overall level of BMI and the overall rate of change in BMI to avoid possible confounding between the RMSE and the overall level and trend in BMI. However, our use of separate trends for positive vs negative overall weight change appears to be novel.

### Strengths and Limitations

Our study has several strengths. One strength was the statistical methods used to estimate residual variation in baseline BMI. Second, anthropometric data were measured in a clinical setting rather than based on participant recall; self-reported anthropometric data can result in overestimation of risk.^37^ Third, we excluded persons with confirmed diagnoses of CVD or cancer during the baseline period, addressing a concern of Casazza et al^33^ regarding confounding by health status (such as that related to unintentional weight loss) in observational studies of weight cycling and mortality. Nevertheless, there may be other, unknown or unmeasured, causes of weight change that are associated with mortality risk; as with any observational study, cautious interpretation regarding causality is required. Fourth, we used continuous BMI rather than BMI categories, thereby avoiding potential artifacts with the somewhat arbitrary selection of a reference BMI category.^38^

There are potential limitations of our study. First, our choice of the degree of polynomial in the model for baseline BMI trajectories could be considered arbitrary. Although the model with a quartic term had the best fit, we deemed it unnecessary to include the fourth-degree term based on inspection of fitted curves and residuals. An LQC model was also used by other authors in a mixed-model analysis of BMI trajectories and weight gain or loss before CVD onset.^39^ Second, reducing weight fluctuation to a single measure (RMSE) does not allow for simple clinical interpretation. Additional information, such as number or magnitude of weight gain and loss cycles, may be useful in studies that focus on prognosis or intervention. Useful measures have been defined by others^39,40^ and might be obtained using time-series or frequency-domain analyses. Third, because mortality information came from death certificates, there may have been some misclassification. A check of cerebrovascular (stroke) death subtypes among the participants revealed approximately equal numbers of cases of cerebral infarction (ischemic stroke) and cerebral hemorrhage. This proportion of cerebral hemorrhage deaths was higher than published incidence rates suggest^41^; thus, we did not examine risk for subtypes of cerebrovascular disease mortality. Fourth, this study was conducted in a Japanese population; results might not apply to other racial/ethnic groups. Few of the participants in our study were obese (BMI>30.0). It is important to understand whether the association between weight fluctuation and mortality differs according to weight class (normal, overweight, or obese). One study^42^ found that weight cycling was associated with sudden cardiac death and heart disease mortality among women who were of normal weight at the time of enrollment but not among women who were overweight or obese. Although we did not observe a significant difference between persons with normal BMI and overweight persons in terms of risk of total mortality, the estimated risk was higher in overweight persons. Fifth, we did not attempt to account for an association between smoking cessation and body weight.^43^ However, it is difficult to distinguish the effects of smoking on body weight and associated conditions.^44^ Sixth, we did not assess whether weight changes were intentional. However, the primary concern was that unintentional weight loss may have reflected underlying diseases that are associated with increased mortality risk,^45^ and as mentioned above, it is a strength of our study that we were able to identify and exclude participants with known CVD or cancer during the period in which weight change was measured. Seventh, we did not measure adiposity or body fat distribution directly. Although BMI is an imperfect measure of adiposity, BMI and body fat percentage are independently associated with mortality,^46^ both are associated with risk of heart failure,^47^ and obesity increases risk of ischemic heart disease independently of lack of physical fitness.^48^ Body fat distribution has been reported to be a better measure of colorectal cancer risk than BMI,^49^ but overall adiposity is relevant to CVD risk.

## Conclusions

By using a novel statistical approach that removed variation attributable to simple nonlinear changes in weight, our study found an association between weight variation and risk of CVD (except for cerebrovascular disease) mortality and greater risk than has been reported previously to date. To further address concerns about whether it is prudent to recommend that overweight or obese adults lose weight given that attempts at weight loss frequently result in weight cycling, we recommend considering all health aspects of weight loss and weight cycling and articulating better approaches to quantifying and defining weight cycling from a statistical perspective. If weight loss is considered to be important for overall health, we suggest developing methods of intervention that can better ensure successful weight loss.

## Notes

Supplement. **eTable 1.** ICD codes for ischemic heart disease and stroke **eTable 2.** ICD codes for cardiovascular-disease and cancer causes of death **eTable 3.** BMI distributions according to whether or not participants attended at least seven exams during the baseline period **eTable 4.** Distribution of city and sex among participants eligible for follow-up analysis, according to whether they were used or not **eTable 5.** Demographic profiles of individual causes of death **eFigure 1.** Flowchart illustrating participant selection. **eFigure 2.** Age at death among study participants who were eligible for the follow-up analysis, by whether they were used in the follow-up analysis or not **eFigure 3.** Observed longitudinal values of BMI in randomly selected participants, separated by quintile of residual BMI root mean squared error (RMSE) **eFigure 4.** Survival plots for total mortality based on residual BMI root mean squared error (RMSE) quintiles **eFigure 5.** Relative risk of total mortality in twenty equal-sized groups of residual BMI root mean squared error (RMSE) **eMethods.** Statistical models
