# Supplementary material for: Association of Weight Fluctuation With Mortality in Japanese Adults
Source: JAMA Netw Open. 2019 Mar 15;2(3):e190731. doi: 10.1001/jamanetworkopen.2019.0731 (PMC6484619; doi:10.1001/jamanetworkopen.2019.0731)
Supplement: Supplement. — eTable 1. ICD codes for ischemic heart disease and stroke eTable 2. ICD codes for cardiovascular-disease and cancer causes of death eTable 3. BMI distributions according to whether or not participants attended at least seven exams during the baseline period eTable 4. Distribution of city and sex among participants eligible for follow-up analysis, according to whether they were used or not eTable 5. Demographic profiles of individual causes of death eFigure 1. Flowchart illustrating participant selection. eFigure 2. Age at death among study participants who were eligible for the follow-up analysis, by whether they were used in the follow-up analysis or not eFigure 3. Observed longitudinal values of BMI in randomly selected participants, separated by quintile of residual BMI root mean squared error (RMSE) eFigure 4. Survival plots for total mortality based on residual BMI root mean squared error (RMSE) quintiles eFigure 5. Relative risk of total mortality in twenty equal-sized groups of residual BMI root mean squared error (RMSE) eMethods. Statistical models [file jamanetwopen-2-e190731-s001.pdf]

Supplementary Online Content

Cologne J, Takahashi I, French B, et al. Association of weight fluctuation with mortality in Japanese adults. *JAMA Netw Open.* 2019;2(3):e190731. doi:10.1001/jamanetworkopen.2019.0731

eTable 1. ICD codes for ischemic heart disease and stroke

eTable 2. ICD codes for cardiovascular-disease and cancer causes of death

eTable 3. BMI distributions according to whether or not participants attended at least seven exams during the baseline period

eTable 4. Distribution of city and sex among participants eligible for follow-up analysis, according to whether they were used or not

eTable 5. Demographic profiles of individual causes of death

eFigure 1. Flowchart illustrating participant selection.

eFigure 2. Age at death among study participants who were eligible for the follow-up analysis, by whether they were used in the follow-up analysis or not

eFigure 3. Observed longitudinal values of BMI in randomly selected participants, separated by quintile of residual BMI root mean squared error (RMSE)

eFigure 4. Survival plots for total mortality based on residual BMI root mean squared error (RMSE) quintiles

eFigure 5. Relative risk of total mortality in twenty equal-sized groups of residual BMI root mean squared error (RMSE)

eMethods. Statistical models

This supplementary material has been provided by the authors to give readers additional information about their work.

**eTable 1. ICD codes for ischemic heart disease and stroke**				
	**ICD-7**	**ICD-8**	**ICD-9**	**ICD-10**
Ischemic heart disease (IHD)	420	410-414	410-414	I20,21,23-25
Stroke				
(1) Hemorrhage	331	431	431	I61
(2) Infarction	332	432-434	433, 434	I63,65,66
(3) Unclassifiable	334	438	437	I64
(4) Other	334, 352	438	437	I63
(5) Sequelae of cerebrovascular disease	352	-	438	I69 (excluding I698)

eTable 2. ICD codes for cardiovascular-disease and cancer causes of death				
	**ICD-7**	**ICD-8**	**ICD-9**	**ICD-10**
Ischemic heart disease	420	410-414	410-414	I20-I25
Cerebro-vascular disease	330-334	430-438	430-438	I60-I69
All cardio-vascular disease	330-334, 400-416, 420-434, 440-447, 450-468	390-458	390-459	I00-I99
Cancer	ICD0 140-199.9 (excluding benign neoplasms)			

eTable 1 lists the ICD codes used to define heart disease and stroke for exclusion of participants during the baseline period. Participants were excluded whether the diagnosis was acceptable or questionable, so even participants with uncertain (but suspected) diagnoses of heart disease or stroke were excluded.

eTable 2 lists the ICD codes used to define individual causes of death from death certificates.

eTable 3 BMI distributions according to whether or not participants attended at least seven exams during the baseline period

Cycle	Attended seven or more exams						Attended fewer than seven exams				
	No.	Mean	SD	Min	Max		No.	Mean	SD	Min	Max
1	4,446	21.21	2.75	12.0	44.1		2,362	21.28	3.05	12.6	42.3
2	4,517	21.22	2.85	13.5	41.2		1,978	21.43	3.07	14.2	37.6
3	4,619	21.35	2.96	14.2	38.5		1,778	21.65	3.17	13.3	38.8
4	4,675	21.64	3.10	13.9	39.3		1,554	21.99	3.18	14.2	43.9
5	4,686	21.92	3.19	14.7	40.5		1,325	22.24	3.23	14.2	37.6
6	4,696	22.17	3.26	13.9	42.7		1,129	22.45	3.19	13.9	37.7
7	4,702	22.41	3.30	14.2	42.7		906	22.62	3.10	12.5	36.1
8	4,625	22.49	3.32	13.2	42.7		824	22.60	3.02	14.0	36.1
9	4,593	22.56	3.33	13.4	42.7		761	22.65	3.09	14.5	34.9
10	4,512	22.67	3.36	12.5	42.7		692	22.76	3.14	14.0	35.1

eTable 3 shows average BMI values for all eligible participants (cohort members who attended at least one exam during the baseline period, were of age 20 through 49 at the first baseline exam, survived throughout the baseline period, and had no recorded diagnosis of cardiovascular disease or cancer during the baseline period) at each cycle of examination during the baseline period, according to whether they were included in the final baseline sample (attended seven or more exams) or not (attended fewer than seven exams). There are no noteworthy differences in BMI mean or variation, although the number of participants ("No.") with fewer than seven exams declines (as expected) due to dropping out for reasons other than death. Some of those who dropped out might have suffered from cardiovascular disease events that were not recorded due to lack of attendance at exams.

© 2019 Cologne J et al. *JAMA Network Open.*

eTable 4. Distribution of city and sex among participants eligible for follow-up analysis, according to whether they were used or not

NOT USED (did not attend any exams during the follow-up period)

Sex	City: Hiroshima	Nagasaki	Total
Men	46	13	59
Row %	77.97	22.03	100.00
Column %	34.59	37.14	35.12
Women	87	22	109
Row %	79.82	20.18	100.00
Column %	65.41	62.86	64.88
Total	133	35	168
Row %	79.17	20.83	100.00
Column %	100.00	100.00	100.00

USED (attended at least one exam during the follow-up period)

Sex	City: Hiroshima	Nagasaki	Total
Men	786	411	1,197
Row %	65.66	34.34	100.00
Column %	30.55	34.08	31.68
Women	1,787	795	2,582
Row %	69.21	30.79	100.00
Column %	69.45	65.92	68.32
Total	2,573	1,206	3,779
Row %	68.09	31.91	100.00
Column %	100.00	100.00	100.00

eTable 4 shows the distribution of sex and city of residence according to whether eligible participants were used in the follow-up analysis or not (they were not used if they never attended a clinic exam during the follow-up period).

© 2019 Cologne J et al. *JAMA Network Open.*

eTable 5. Demographic profiles of individual causes of death

Cause of death	Hiroshima men		Hiroshima women		Nagasaki men		Nagasaki women		Total		Grand total
	Never smokers	Ever smokers	Never smokers	Ever smokers	Never smokers	Ever smokers	Never smokers	Ever smokers	Never smokers	Ever smokers	
All causes	25	380	557	151	20	187	177	53	779	771	1,550
Ischemic heart disease	2	19	27	9	0	10	13	2	42	40	82
Cerebrovascular disease	7	31	71	21	2	16	26	7	106	75	181
Other cardio-vascular disease	1	33	89	20	4	15	16	8	110	76	186
Cancer	7	190	187	55	5	81	70	20	269	346	615
Other causes	8	107	183	46	9	65	52	16	252	234	486

© 2019 Cologne J et al. *JAMA Network Open*.

eFigure 1. Flowchart illustrating participant selection.

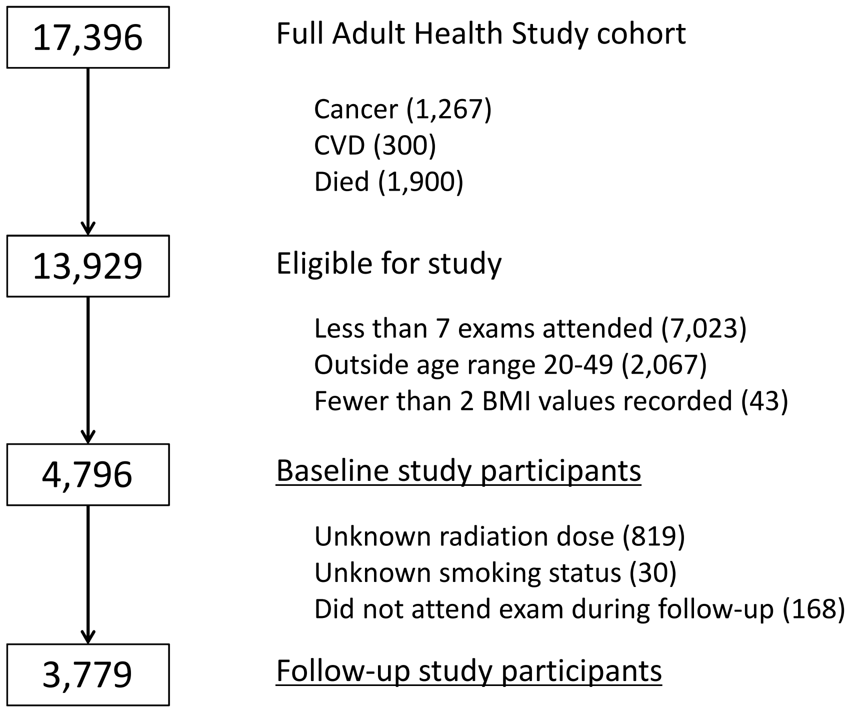

© 2019 Cologne J et al. *JAMA Network Open.*

eFigure 2. Age at death among study participants who were eligible for the follow-up analysis, by whether they were used in the follow-up analysis or not

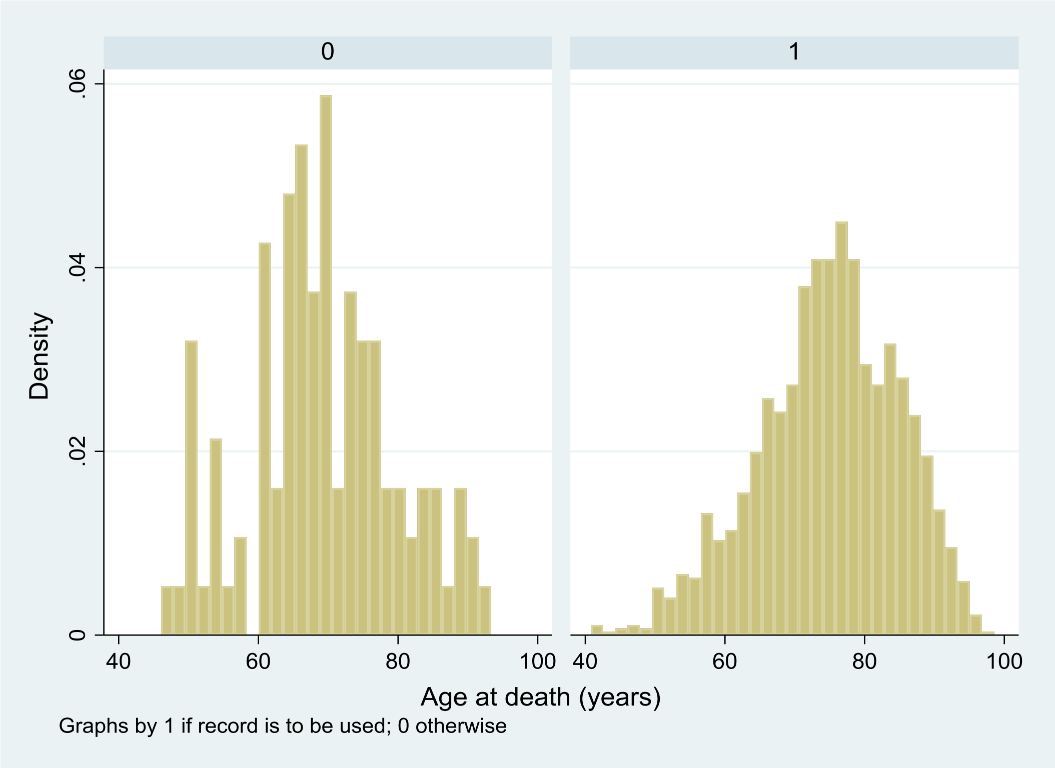

eFigure 2 shows the distribution of ages at death among the eligible study participants, stratified on whether they were used in the follow-up analysis ("1": attended at least one clinic exam during the follow-up period; 3,779 individuals) or not ("0": 168 individuals).

© 2019 Cologne J et al. *JAMA Network Open.*

eFigure 3. Observed longitudinal values of BMI in randomly selected participants, separated by quintile of residual BMI root mean squared error (RMSE)

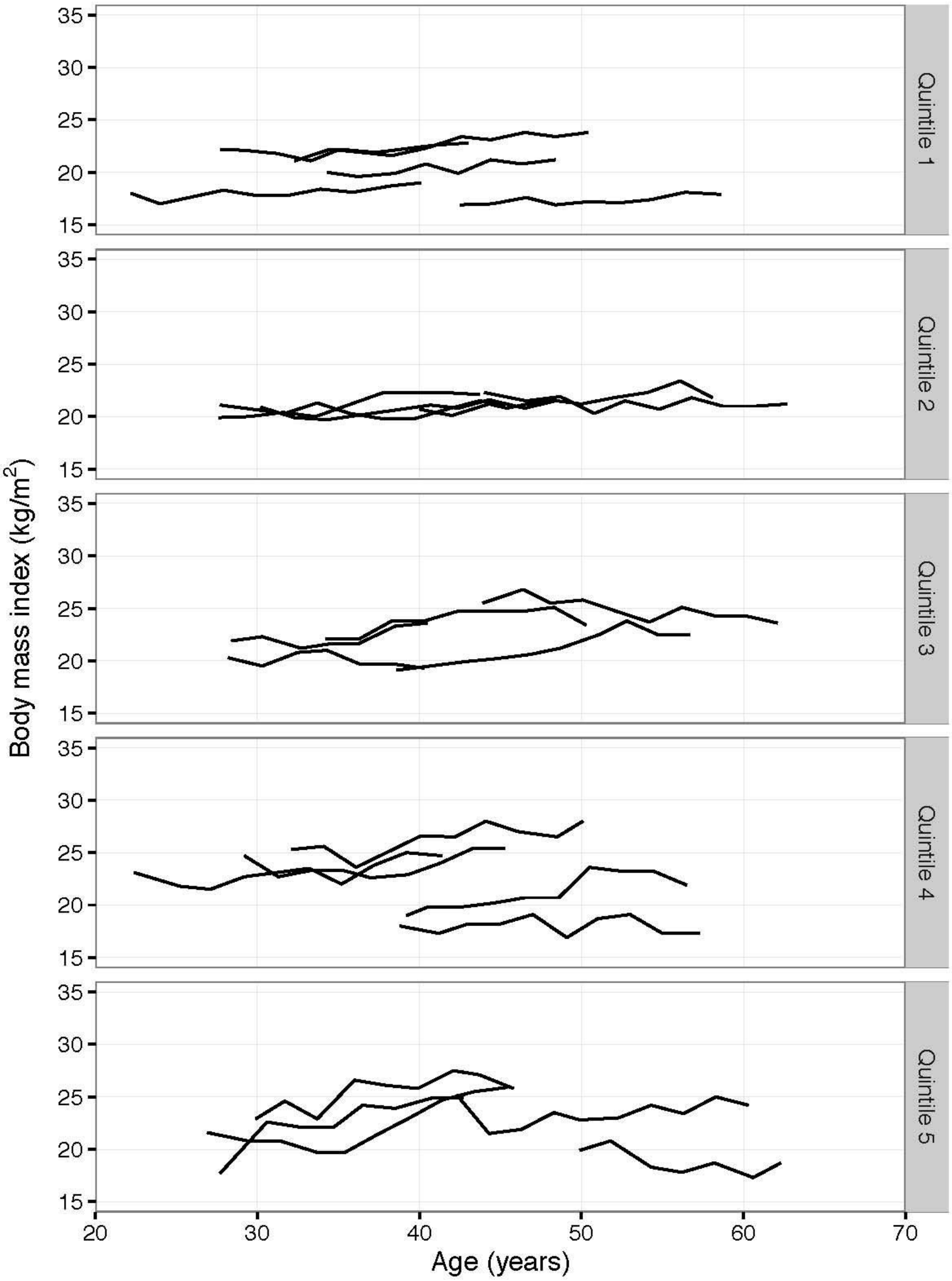

eFigure 3 shows observed BMI trajectories for five randomly selected participants in each of the five RMSE quintiles (quintile 1 is the lowest, quintile 5 is the highest). Each line represents the BMI trajectory of one participant selected at random. An increase in within-individual variation or fluctuation with increasing RMSE is apparent.

© 2019 Cologne J et al. *JAMA Network Open.*

eFigure 4. Survival plots for total mortality based on quintile of residual BMI root mean squared error (RMSE)

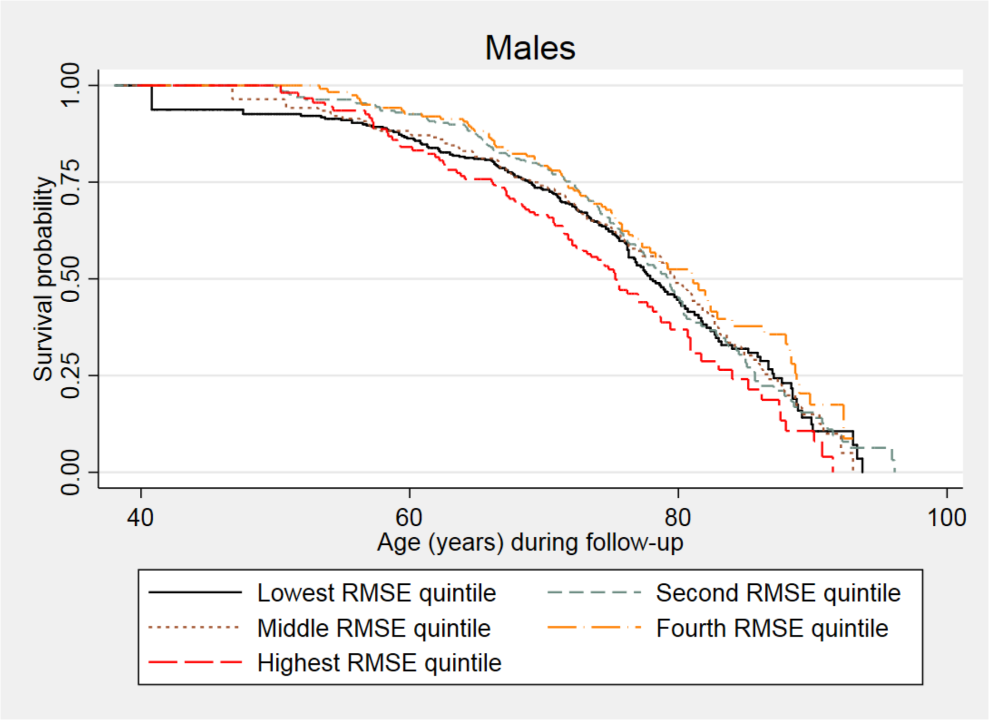

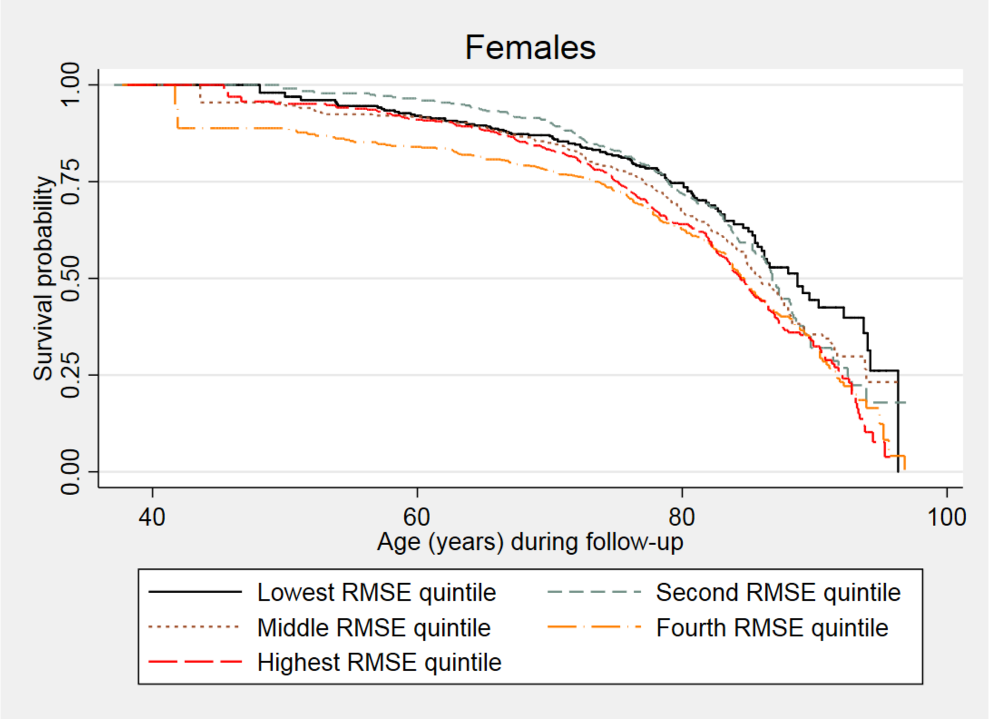

eFigure 4 shows crude survival probability by RMSE quintile separately for men and women. Solid line is the lowest quintile, short dashes represent the 2nd quintile, dots represent the middle quintile, dashes & dots represent the 4th quintile, and long dashes represent the highest quintile. Men in the highest quintile had lower survival probability throughout most of the follow-up period. Women in the highest quintile had lower survival

© 2019 Cologne J et al. *JAMA Network Open.*

probability in the early follow-up period, and had survival probability no better than that of other RMSE groups during the latter follow-up period.

© 2019 Cologne J et al. *JAMA Network Open.*

eFigure 5. Relative risk of total mortality in twenty equal-sized groups of residual BMI root mean squared error (RMSE)

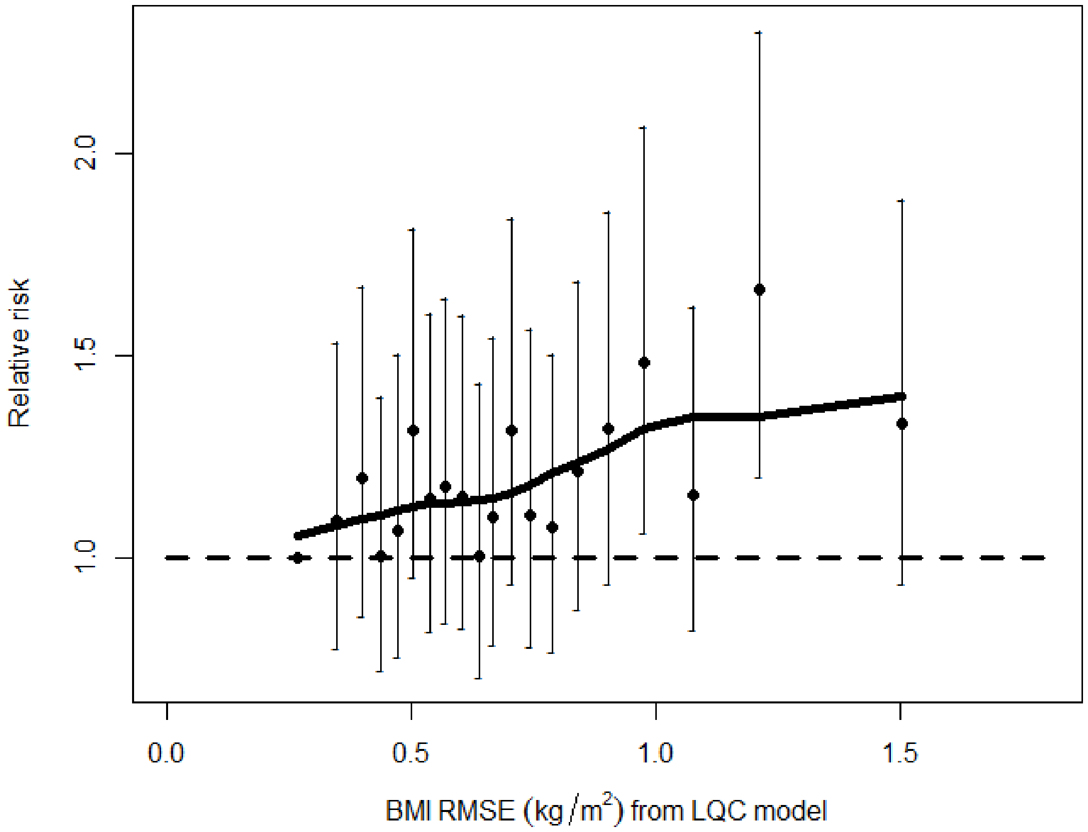

Relative risk (RR, with 95% CI) of total mortality was obtained by using the same Cox model as in Table 4 of the manuscript, except that 20 equal-sized groups of RMSE were used rather than quintiles. The lowest group was used as the reference. The line is the fit of a lowess smooth curve with fraction parameter 0.75. The lowess curve reveals a nearly linear increase in risk with increasing RMSE. The RR at 1.0 kg/m^2 obtained from the fitted curve is close to the log-linear relative risk estimate of 1.27 from manuscript Table 3. Note that these confidence intervals reflect small numbers of participants, so no meaning can be attached in terms of statistical significance; what is important is that the nonparametric smooth curve supports the finding of a significant increase in risk with increasing residual variation in BMI, as in manuscript Table 3.

© 2019 Cologne J et al. *JAMA Network Open.*

eMethods. Statistical models

The mixed effects (random-coefficient) model used to estimate smooth baseline BMI trajectories is:

$$\text{Level 1 model} \quad Y_{ij} = \delta_{01} + \delta_{i1}a_{ij} + \delta_{i2}a_{ij}^2 + \delta_{i3}a_{ij}^3 + \beta_1 s_i + \beta_2 a_{i0} + \beta_3 e_i + \varepsilon_{ij}$$

$$\text{Level 2 model} \quad \begin{cases} \delta_{01} = \gamma_{00} + \varsigma_{0i} \\ \delta_{i1} = \gamma_{10} + \gamma_{11}s_i + \varsigma_{1i} \\ \delta_{i2} = \gamma_{20} + \varsigma_{2i} \\ \delta_{i3} = \gamma_{30} + \varsigma_{3i} \end{cases} \qquad \text{(e1)}$$

where a_{ij} is longitudinal age of participant i at baseline time j (centered at 44 y), s_i is the sex of subject i (coded 0 for males, 1 for females), and a_{i0} is age at entry (centered at 35 y), with $\varepsilon_{ij} \sim N(0,\sigma_\varepsilon^2)$ and $\boldsymbol{\varsigma} \sim N_4\left(\boldsymbol{0}, \text{diag}\left\{\sigma_{\varsigma_k}^2\right\}\right)$ ("~" denotes "is distributed as"). We also assessed the effect of radiation dose (e_i) as a further predictor in the level 1 model, as noted in the methods section of the main paper, but radiation dose was not retained in the final analysis as it had no effect. The level 1 model specifies how BMI depends on age (i.e. a polynomial model) for a given subject, as well as how BMI depends on other covariates that are fixed within each subject (age at entry into the study and sex). The level 2 model specifies the random effects (coefficients of the longitudinal BMI trajectory), allowing for those coefficients to vary among subjects. The linear random-coefficient model used to derive overall BMI level and slope random effects is model (e1) with only the first two terms in the level 2 model.

The basic Cox model used for estimating risk for residual BMI variability is:

$$\lambda(t) = \lambda^0(t; c \times s)\exp\left\{\alpha_1\tau + \alpha_2 b + \alpha_3 h + \alpha_4 w + \alpha_5 e + \alpha_6 d + \alpha_7 g^+ + \alpha_8 g^-\right\} \qquad \text{(e2)}$$

where $\lambda(t)$ is the rate of mortality at time t (age) during the follow-up period with left truncation on age at start of follow-up, $\lambda(t;c\times s)$ is the background mortality function for city-sex stratum $c\times s$, τ is residual root mean squared error (RMSE) from the LQC two-level model for BMI, b is year of birth (centered at 1924), h is body height averaged over the baseline period (centered at 161.5 cm for males and 160.0 cm for females), w is the random effect for the intercept from a two-level linear model for BMI (subject-specific residual for overall level of BMI during the baseline period), e is an indicator of having ever smoked, d is DS02R1 radiation dose to the colon (gy;

© 2019 Cologne J et al. *JAMA Network Open.*

not centered, as zero is the natural reference value), g^{+} is the random effect for the slope from a two-level linear model for BMI if positive, and g^{-} is that random effect for the linear-model slope if negative (overall increase or decrease, respectively, compared with the population slope). For reference, the mean (median) values of the random-effects for intercept and slope increase or decrease were 0.017 (−0.344), 0.440 (0.0), and 0.442 (0.077), respectively. Note that the last value is the magnitude of decrease in slope, so the actual mean random effect among persons who had BMI loss overall is −0.442.
